# The Next-Generation Sequencing Quality Initiative and Challenges in Clinical and Public Health Laboratories 

**DOI:** 10.3201/eid3113.241175

**Published:** 2025-05

**Authors:** Blake Cherney, Ariel Diaz, Camby Chavis, Christopher Ghattas, Diana Evans, Diego Arambula, Heather Stang, on behalf of the Next-Generation Sequencing Quality Initiative

**Affiliations:** Author affiliations: Centers for Disease Control and Prevention, Atlanta, Georgia, USA (B. Cherney, A. Diaz, C. Chavis, C. Gattas, D. Evans, D. Arambula, H. Stang); Booz Allen Hamilton Inc., Atlanta (A. Diaz, C. Chavis, C. Ghattas, D. Evans)

**Keywords:** next-generation sequencing, NGS, QMS, validation, public health laboratories, CLIA, quality management system, CDC, Association of Public Health Laboratories

## Abstract

The Next-Generation Sequencing (NGS) Quality Initiative addresses laboratory challenges faced when performing NGS by developing tools and resources to build a robust quality management system. Here, we illustrate how those products support laboratories in navigating complex regulatory environments and quality-related challenges while implementing NGS effectively in an evolving landscape.

Next-generation sequencing (NGS) technology has improved with the introduction of new platforms, updated chemistries, advancements in bioinformatic analyses, and computational innovations. As targeted and agnostic (e.g., metagenomic) sequencing approaches have been introduced, validation of NGS assays has increased in complexity, mostly because of sample type variability, stringent quality control criteria, intricate library preparation, and evolving bioinformatics tools (*1**–**3*). Complexity increases when validations are governed by the Clinical Laboratory Improvement Amendments of 1988 (CLIA) (*4*).

Performing NGS requires an experienced workforce to generate high-quality results. Retaining proficient personnel can be a substantial obstacle because of the unique and specialized knowledge required of them, which in turn increases costs for adequate staff compensation. Akkari et al. (*5*) found that some testing personnel held their positions for <4 years on average. In 2021, the Association of Public Health Laboratories (APHL) reported that 30% of surveyed public health laboratory staff indicated an intent to leave the workforce within the next 5 years (*6*). Additional barriers may arise when hiring and qualifying personnel under regulations such as CLIA and state hiring statutes (*4*).

## The Study

**Figure 1:**
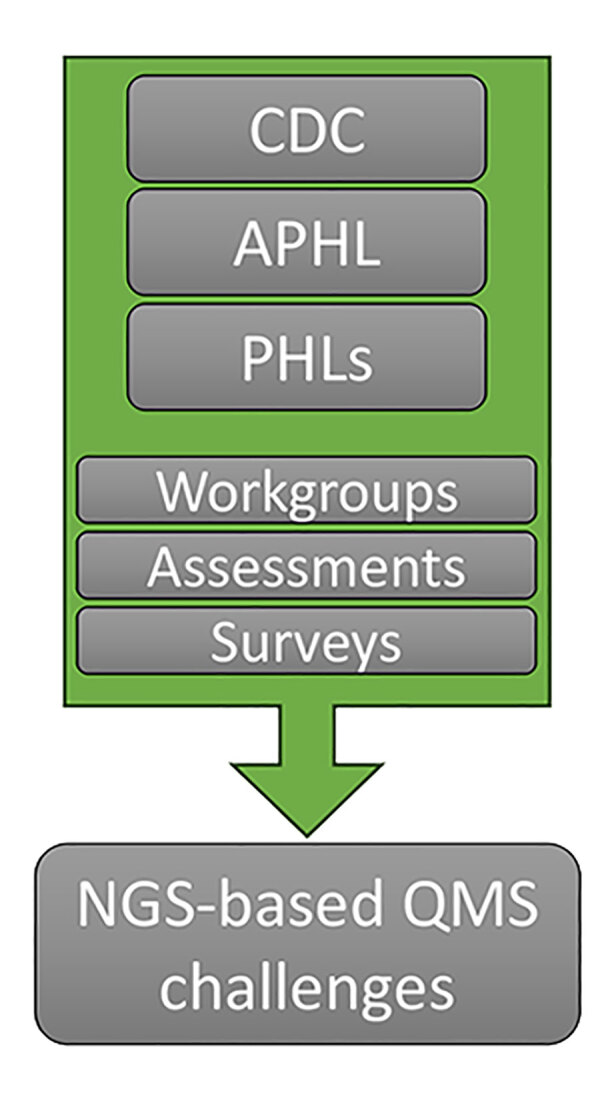
Partners and aim of the Next-Generation Sequencing Quality Initiative. Partners collaborate to identify and address NGS-specific challenges through development of a QMS. APHL, Association of Public Health Laboratories; CDC, Centers for Disease Control and Prevention; NGS, next-generation sequencing; PHL, public health laboratories; QMS, quality management system.

**Figure 2:**
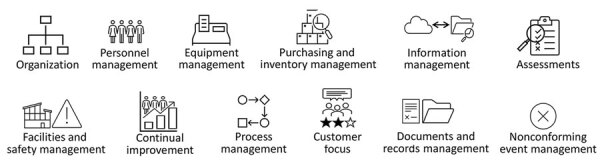
Depiction of Clinical and Laboratory Standards Institute’s 12 QSEs as building blocks for tools and documents available on the website (https://www.cdc.gov/lab-quality/php/ngs-quality-initiative/qms-tools-resources.html). QSE, Quality System Essentials.

In an effort to help clinical and public health laboratories, the Centers for Disease Control and Prevention and APHL collaborated to form the Next-Generation Sequencing Quality Initiative (NGS QI; https://www.cdc.gov/lab-quality/php/ngs-quality-initiative/index.html) to address challenges associated with implementing NGS in clinical and public health settings. NGS QI staff performed an initial assessment of needs and identified common challenges associated with personnel management, equipment management, and process management across NGS laboratories (Figure 1; Appendix). Among those challenges was a lack of high-quality guidance documents and standard operating procedures (SOPs) (*7*). The NGS QI found that laboratories were developing in-house resources that, although similar in content, contained varying levels of detail (*7*,*8*). The Initiative provides publicly available tools that can be used regardless of platform, agent, or application and that satisfy the needs of laboratories whether they are implementing NGS initially or refining existing workflows (Figure 2; Appendix).

**Figure 3:**
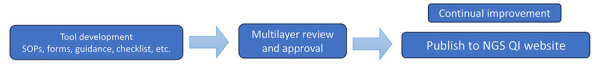
Depiction of the review and approval process for tools and documents published on the QI website (https://www.cdc.gov/lab-quality/php/ngs-quality-initiative/qms-tools-resources.html). NGS, Next-Generation Sequencing; QI, quality initiative; SOP, standard operating procedures.

A quality management system (QMS) enables continual improvement and proper document management in laboratories. All existing NGS QI products undergo a review period every 3 years to ensure they remain up to date relative to current technology, standard practice of care, and applicable changes in regulations (Figure 3). Previous internal surveys and workgroups identified validation tools as a high-priority task to assist laboratories in ensuring compliance with quality and regulatory standards (*9*). In response, the NGS QI created the Pathway to Quality-Focused Testing; although it is not a standalone document for developing an NGS-specific QMS, it complements other published tools and resources that address relevant topics in depth. Other recently released documents are tailored to validation and bioinformatic development, ranging from straightforward guidance to fillable templates. Since its establishment, the NGS QI has seen an increasing interest in NGS method validation because most clinical and public health laboratories are already using or are beginning to implement NGS within their workflows. For that reason, many of the NGS QI’s resources assist with NGS assays for validation. The most widely used documents offered by the NGS QI are QMS Assessment Tool, Identifying and Monitoring NGS Key Performance Indicators SOP, NGS Method Validation Plan, and the NGS Method Validation SOP (Table). For example, the use of the Validation Plan document guided Orange County Public Health Laboratory (Santa Ana, California, USA) in generating a standard template containing NGS-related metrics, thereby reducing the burden on laboratories seeking to perform a validation (*10*).

**Table:** Most frequently downloaded documents of the 113 posted on the Next-Generation Sequencing Quality Initiative website during January–June 2024*

Document	No. views
QMS Assessment Tool	548
Identifying and Monitoring NGS Key Performance Indicators SOP	410
NGS Method Validation Plan	410
NGS Method Validation SOP	199

*Total of 11,790 visits to website (https://www.cdc.gov/lab-quality/php/ngs-quality-initiative/qms-tools-resources.html) during July 2023–July 2024. NGS, next-generation sequencing; QMS, quality management system; SOP, standard operating procedure;

The NGS QI develops and crosswalks its documents with regulatory, accreditation, and professional bodies (e.g., the US Food and Drug Administration [FDA], Centers for Medicare and Medicaid Services, and College of American Pathologists) to ensure they provide current and compliant guidance on Quality System Essentials (QSE) (Figure 3; Appendix) (*4*,*11*). To support challenges associated with staff training and competency assessment, the NGS QI has published 25 tools for the personnel management QSE (e.g., Bioinformatics Employee Training SOP) and 4 tools for the assessments QSE (e.g., Bioinformatician Competency Assessment SOP); the Initiative also works with partners to host or participate in online trainings (Appendix). A QMS must be able to adapt to an ever-changing environment, including improvements in software and chemistry, which can affect how validated NGS assays, pipelines, and results are developed, performed, and reported. Even as laboratories become more familiar with guidance documents and standard practices, there are other challenges: information technology cost, curated databases, developing standards, and newer platforms. For example, new kit chemistries from Oxford Nanopore Technologies (https://nanoporetech.com) that use CRISPR for targeted sequencing and improved basecaller algorithms using artificial intelligence, machine learning, and duplex data lead to increased accuracy (*2*). Other emerging platforms, such as Element Biosciences (https://www.elementbiosciences.com), also show increasing accuracies at Q40 with lower costs, which might encourage transition from older platforms to new platforms and chemistries (*12*). Although modernizing is beneficial, transitioning to new platforms requires additional resources and time to revalidate NGS workflows. Changes in policies and regulations can also create confusion and barriers for laboratories (*13*). 

## Conclusion

**Figure 4:**
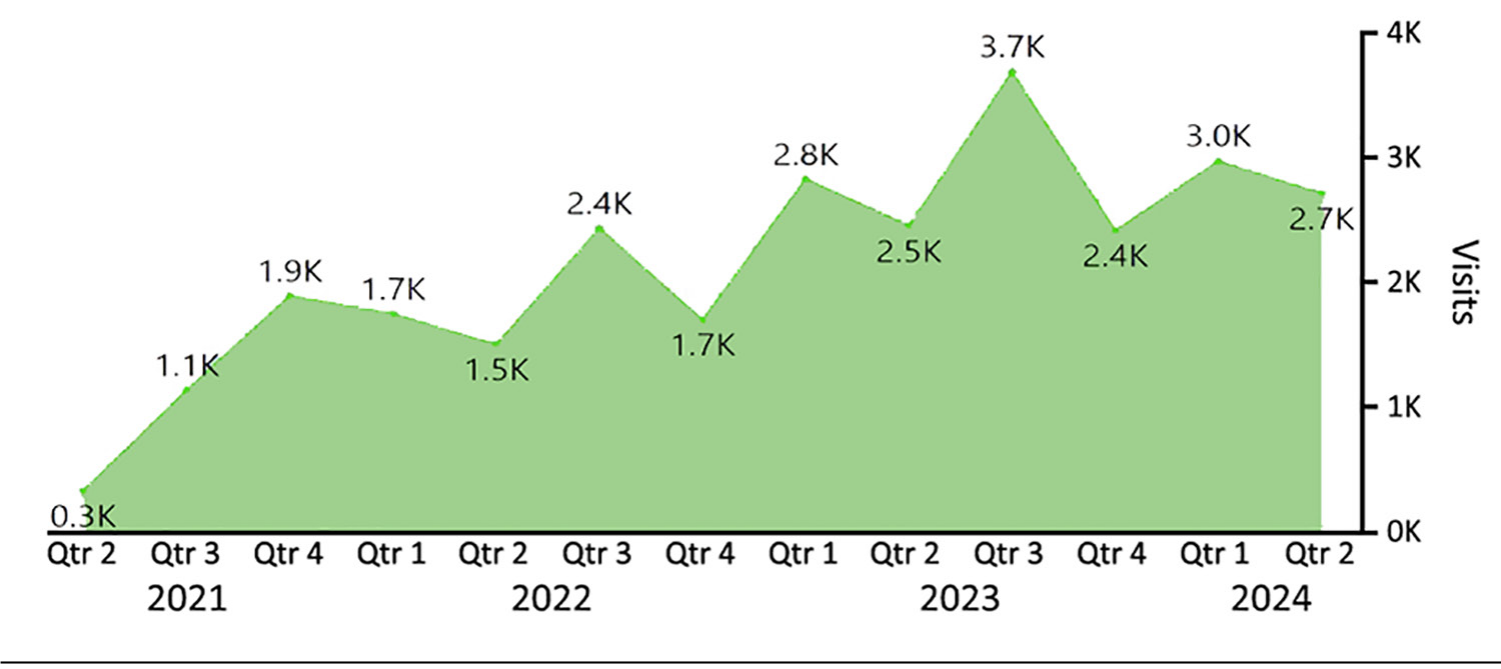
Trends in visits to the Next-Generation Sequencing Quality Initiative website (https://www.cdc.gov/lab-quality/php/ngs-quality-initiative/qms-tools-resources.html), by quarter, 2021–2024.

NGS is complex, and workflows often differ among specialties and sequencing approaches. Despite advancements in guidance, practice, and technology, NGS validation remains challenging. The NGS QI generates resources that are written broadly enough to benefit an array of laboratories and methods. Limitations in regulatory authority often prevent the development of prescriptive guidance. Although NGS QI’s tools are applicable to most platforms and applications, the laboratories using each product may have additional quality assurance considerations. To keep up with evolving practices, the Initiative conducts cyclic review and performs regular or ad hoc (if significant changes warrant) updates. However, the rapid pace of changes in policy and technology means that regular updates do not always resolve challenges. Although completing a method validation or revalidation is resource intensive, it is important that, once validated, the entire workflow is locked down (*13*). Evaluating technological advancements is necessary; the shifts in testing needs for patient populations, the evolving public-health applications, and the ability to modify sequencing workflows depend heavily on institutional practices and regulatory bodies (i.e., local, state, federal, and accrediting organizations). Those factors indicate the need for bespoke practices among entities. On the path of creating high-quality, reproducible, and reliable results, obstacles will continuously arise. It is imperative to use a balanced review process to implement changes to sequencing workflows and stay current relative to the latest advancements, best practices, and regulatory requirements, which may not always align for practical implementation. As the pool of NGS QI’s users continues to grow (Figure 4), the Initiative will continue adapting to needs by creating supporting documents and trainings focused on the application of the NGS QI documents, tools for emerging challenges (e.g., validation of machine learning algorithms, agnostic pathogen detection), curated databases, clinical decision tools, and frontline diagnostics for clinical and public health laboratories.

Appendix Additional information about the Next-Generation Sequencing Quality Initiative and current and emerging challenges for clinical and public health laboratories.
