# Supplementary material for: The Next-Generation Sequencing Quality Initiative and Challenges in Clinical and Public Health Laboratories
Source: Emerg Infect Dis. 2025 May;31(Suppl 1):S14–7. doi: 10.3201/eid3113.241175 (PMC12078536; doi:10.3201/eid3113.241175)
Supplement: Appendix — Additional information about the Next-Generation Sequencing Quality Initiative and current and emerging challenges for clinical and public health laboratories. [file 24-1175-Techapp-s1.pdf]

Article DOI: https://doi.org/10.3201/eid3113.241175

EID cannot ensure accessibility for supplementary materials supplied by authors. Readers who have difficulty accessing supplementary content should contact the authors for assistance.

The Next-Generation Sequencing Quality Initiative and Challenges for Clinical and Public Health Laboratories

Appendix

NGS QI Process Overview

The Next-Generation Sequencing (NGS) Quality Initiative (QI) works with diverse federal and non-federal partners, which include the NGS QI Leadership Team, representative from the Association of Public Health Laboratories (APHL), members of the Centers for Disease Control and Prevention (CDC) NGS Quality Workgroup (QWG), the Technical Coordinating Committee for the Advanced Molecular Detection (AMD) Platform and NGS QI (TANQ), and partner state and local public health laboratories (PHLs), meet regularly to discuss persistent and emerging, quality-related sequencing challenges. The NGS QI published an NGS-focused, foundational quality management system (QMS) that was designed to be easily incorporated into existing laboratory quality practices or to serve as a stand-alone foundational system. The NGS QI bases its work on the Clinical & Laboratory Standards Institute's (CLSI) framework of 12 Quality Systems Essentials (QSEs): Personnel Management, Equipment Management, Process Management, Information Management, Documents and Records Management, Assessments, Continual Improvement, Facilities and Safety Management, Supplier and Inventory Management, Nonconforming Event Management, Organization and Leadership, and Customer Focus. Where gaps or needs are identified, the NGS QI develops relevant products and resources to satisfy said needs. All efforts are made to ensure that products being developed are not already existing or to incorporate existing work into new product development. A proposal to develop a new product (document, checklist, app, webpage, etc.) originates from a variety of sources, but the CDC NGS QI Leadership Team's approval serves to initiate the development process and is

required for creation of any new product. Once drafted, the new document or tool goes through a structured review and approval process that involves several internal and external review rounds, each with specific timelines. The development process includes a dedicated document owner who oversees development and works with NGS QI specialists and other subject matter experts (SMEs). Each product has its own development timeline. Once product development is agreed upon by the product owner, specialists, and SMEs, products are reviewed by CDC NGS QI Leads (dedicated personnel within the Leadership Team), CDC NGS QWG, PHL SMEs, and TANQ members. Next, the product is routed for CDC clearance, which consists of scientific review by various Centers/Institutes/Offices, communication specialists, and other SMEs (identified as necessary). Prior to posting to the public webpage, all products are made Section 508 compliant to comply with federal law. Typically, products undergo a minimum of four rounds of review by various SMEs before receiving final approval. In addition, published tools undergo constant cyclic review to ensure alignment with current standards and updates in policies, technology, and manufacturer-specific criteria. The NGS QI works alongside federal and non-federal partners (e.g., PHLs) to identify quality-related gaps, to seek out existing resources, and to develop practical products that best address any identified challenges. Below is a flowchart representing the NGS QI document review process (Appendix Figure).

The NGS QI Instructions

Instructions per NGS QI Web site:

1. Click the link: https://www.cdc.gov/lab-quality/php/ngs-quality-initiative/qms-tools-resources.html.
2. The website allows users to sort tools by different categories, including manufacturer, role, NGS Quality Maturity Level, Quality System Essential, and specific queries under the “search for tools” bar.
3. Recently, the NGS QI published the Pathway to Quality-Focused Testing tool, which is intended to support laboratory personnel responsible for validation, continued testing, and maintenance of NGS workflows. To access, please use the following link: https://www.cdc.gov/lab-quality/php/pathway/pathway-to-testing.html.

4. If you would like to convey NGS-Based QMS needs for your laboratory, share tools (e.g., SOPs, forms, guidance, etc.), or report any technical issues, please send an email to NGSQuality@cdc.gov.

C. NGS QI's Examples Supporting Laboratories and Impact

The NGS QI has supported the implementation of tools for the Clinical Laboratory Improvement Amendments (CLIA) Laboratories and APHL; specific examples include:

1. CDC CLIA Compliance Program is currently using the Pathway to Quality-Focused Testing tool to support laboratory personnel and validation. Dr. Jeronay Thomas, Health Scientist of the CDC CLIA Compliance Program, commented: "The NGS QI has significantly enhanced the CLIA program by providing expert review of NGS-related method validation plans and developing resources and pathways that support CLIA personnel in creating robust NGS-related procedures, facilitating the development and implementation of CLIA-compliant NGS assays."
2. The NGS QI developed a training to maximize the use and customization of the QMS Assessment Tool according to specific QMS requirements and metrics. This tool allows laboratories to assess and score an entire QMS by providing quarterly graphs based on specific CLSI's QSEs. More information about the training can be found here: https://www.aphl.org/programs/infectious_disease/Documents/NGSQIAMD_WebinarFlyer_20240919.pdf (October 29, 2024)

During the closing remarks of this webinar, Dr. Lauren Turner, advanced molecular detection lead scientist at Virginia Division of Consolidated Laboratory Services, presented the ways in which these tools have been used by her laboratory and commented that the QMS assessment tool is "an excellent tool for determining needs when creating a new quality assurance system for NGS."

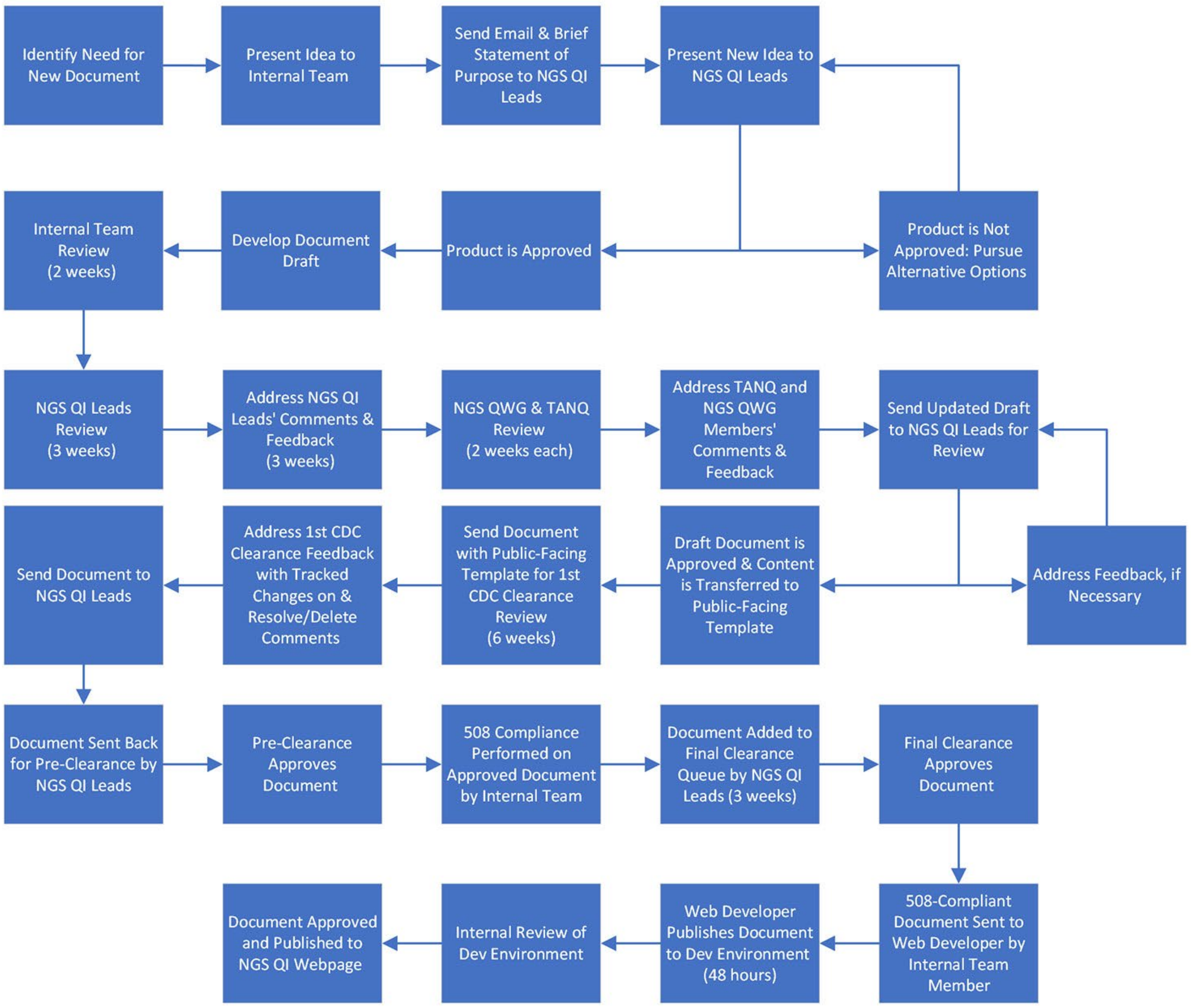

Appendix Figure. Expanded NGS QI process to develop documents and tools.
